# Specialized nurses’ role in ensuring patient safety within the context of telehealth in home care: A scoping review

**DOI:** 10.1177/20552076241287272

**Published:** 2024-10-07

**Authors:** Mojtaba Vaismoradi, John Rae, Hannele Turunen, Patricia A. Logan

**Affiliations:** 1 Faculty of Nursing and Health Sciences, 1786 Nord University, Bodø, Norway; 2 Faculty of Science and Health, Charles Sturt University, Orange, NSW, Australia; 3 Faculty of Science and Health, Charles Sturt University, Bathurst, NSW, Australia; 4 Department of Nursing Science, Kuopio University Hospital, University of Eastern Finland, Kuopio, Finland

**Keywords:** Home care, medication management, patient safety, role, scoping review, specialized nurse, telehealth

## Abstract

**Objectives:**

Specialized nurses are uniquely positioned to implement innovative telehealth solutions to improve the quality and safety of home care, and this has become a focal point of contemporary healthcare research. This review aimed to identify the nature and scope of specialized nurses’ roles in ensuring patient safety within the context of telehealth in home care.

**Methods:**

A scoping review of the international literature was carried out from January 1, 2013, to August 29, 2024. The review employed Levac et al.'s framework to delineate the research phenomenon and consolidate existing empirical research findings. Through a comparative analysis, the review integrated findings from selected studies, highlighting both similarities and differences related to this phenomenon, which led to the development of distinct categories.

**Results:**

The search yielded 1127 articles, from which 23 studies met the inclusion criteria for research synthesis and subsequent reporting of results. These studies spanned specialized nurses’ roles in telehealth and various fields in which specialized nurses utilized telehealth to deliver high-quality and safe home care. The findings highlighted key outcomes linked to the improvement of patient safety in home care encompassing continuity of care, confidence in care, monitoring and early intervention, medication safety, engagement and adherence, and healthcare costs.

**Conclusions:**

The review revealed the crucial role played by specialized nurses in harnessing telehealth in healthcare to meet the highest care standards, creating an environment that prioritizes the well-being and patient safety in home care.

## Introduction

Digital health is the use of technology in healthcare to manage illnesses, reduce health risks, and enhance patient well-being. Telehealth, a form of digital health, employs telecommunications like video conferencing and mobile apps for virtual consultations, remote monitoring, and healthcare support, enabling professionals to assist patients regardless of physical location.^1–3^ It supports chronic disease management by monitoring exacerbations and ensuring continuity of care for those who remain at home.^4^ Telehealth, which has evolved since the 1970s,^5^ experienced a notable uptick in usage during the COVID-19 pandemic^6,7^ and has now attained a status where it is considered an essential requirement for the modern age.^8^

Telehealth solutions have been considered effective in healthcare services in terms of the optimization of patient diagnosis, consultation, and treatment processes^9–11^ and also nurse-led practices such as pain management.^12^ As for patient safety, telehealth has the potential to decrease the occurrence of practice errors^13^ as it mainly focuses on lowering risks, and reducing avoidable patient harm.^14^ For instance, it can support healthcare providers through providing early warning signs to identify the deterioration in the patient condition^15^ and improve care outcomes.^16^ Also, telehealth can enhance the accuracy of information management that is required to increase healthcare providers’ capacity for making correct and well-informed decisions for patient care.^17^

Nurses can play a crucial role in the field of digital health and telehealth given that the quality and safety of healthcare is highly dependent on nurses’ participation and adherence to the principles of patient safety.^18^ Advanced training enables nurses to support themselves and other healthcare professionals in managing patient safety risks, thereby helping prevent patient harm that may lead to moral injury.^19^

The concept of specialized nursing practice encompasses a diverse range of definitions and interpretations, reflecting varied expertise, education, and skills associated with nursing roles, all of which are context sensitive. The Advanced Practice Nursing (APN) guideline by the International Council of Nurses (ICN) highlights two widely recognized roles: the Clinical Nurse Specialist (CNS) and the Nurse Practitioner (NP). These are distinguished by educational and training prerequisites.^20^ A CNS is a nurse having advanced preparation beyond the level of a general nurse and possesses advanced expertise in a specific nursing branch,^21^ whereas an NP assumes the responsibility of integrating clinical skills related to both nursing and medicine in primary and acute healthcare settings.^20^ In the UK, the most used titles are CNS, APN and NP with, variants of seniority and function,^22^ but in the USA, Advanced Practice Registered Nurse (APRN) is common and is referred to CNS and NP.^23,24^ In Canada, advanced nursing practice includes CNS, NP, Primary Healthcare Nurse Practitioners (PHCNPs), and Acute Care Nurse Practitioners (ACNPs), but in Australia specialized nurses are NPs.^24,25^ In Nordic countries, CNS is used for clinical nursing leaders and nurses with high-level clinical competencies.^26^ The definitions and requirements for specialized nursing vary widely across Europe in terms of in titles, education levels, certification, regulation, and scope of practice,^27,28^ but bachelor’s degree nurses generally undergo training for advanced practice and become specialized nurses.^27^

Besides the variations in the titles and responsibilities of specialized nurses, the recognized trait among specialized nurses is an extended set of theoretical and practical competencies in a specific clinical domain, which are shaped by the context in which they are credentialed to practice.^29^ Specialized nurses are empowered to integrate theoretical knowledge and practical abilities, enabling them to take clinical practice, teaching, administration, research, and advisory roles in diverse healthcare environments including home care consisting of managing chronic conditions, providing personalized care, educating patients and families, and coordinating care with healthcare professionals.^20,21,30^ The introduction of specialized nursing offers several advantages for patient safety, including personalized clinical follow-up, evidence-based practice, continuous education for nurses, increased autonomy within their scope of responsibility, and enhanced teamwork. These elements collectively contribute to a more effective and responsive healthcare environment, ensuring high standards of patient care and safety.^31,32^ Emphasizing the competencies of specialized nurses and integrating them into clinical practice is crucial for ensuring high-quality and safe care.^33^

Previous studies have investigated the perceptions and competencies associated with the adoption of telehealth within the healthcare sector, encompassing a range of healthcare professionals. However, there is a gap in the international literature pertaining to the integration of knowledge and the advancement of specialized nurses’ roles in the context of telehealth in home care. This review contributes to a nuanced understanding that can inform targeted strategies, interventions, and educational initiatives tailored to optimize the use of telehealth in specialized nursing practice. Therefore, the aim of this review was to identify the nature and scope of specialized nurses’ roles in ensuring patient safety within the context of telehealth in home care. Accordingly, the review question was: “What is the nature and scope of specialized nurses’ role in ensuring patient safety within the context of telehealth in home care?”

## Methods

### Design

A scoping review was conducted to understand and map the breadth of evidence available^34^ relating to specialized nurses’ roles for ensuring patient safety using telehealth and to elucidate implications for policy making and future research.^35^

The review framework proposed by Levac et al.^36^ based on the Arksey and O’Malley methodology^37^ was employed. This involved taking the following steps: formulating the research question, conducting a literature search, and retrieving pertinent studies, selecting relevant studies, charting, collating and summarizing data, reporting results, and seeking consultation.

### Formulating the research question

The review question was formulated as follows: “What is the nature and scope of the role of specialized nurses in ensuring patient safety within the context of telehealth in home care?” The review's aim and process were established based upon the PICo statement^38^ as follows:

P (Population): Specialized nurses typically holding bachelor's or master's degrees in nursing and receiving additional training and education in a specific field of nursing to be equipped with specialized knowledge and skills to provide focused and expert care within a particular patient population or clinical setting; examples include nurses formally recognized as APN, CNS, NP, and Clinical Nurse Consultant (CNC).

I (Interest): Roles as practical considerations, interventions, and strategies to ensure patient safety in home care practices.

Co (Context): Telehealth like video conferencing, mobile apps, and other digital platforms enabling virtual consultations, remote monitoring, and various healthcare activities, allowing the nurses to offer services, consultations, and support to distant patients living in their own home.

### Conducting a literature search and retrieving relevant studies

The authors developed the review protocol, reaching a consensus on its details (Supplementary File 1). Conducting a pilot search on general databases and drawing from their prior review experiences, they formulated search phrases using keywords, Medical Subject Headings (MeSH), and thesaurus entry terms translatable into scientific databases. Employing the Boolean method and truncations with AND/OR operators, they constructed the search string (Supplementary File 2), which underwent a pilot test to confirm its efficacy in retrieving relevant studies. It encompassed various iterations of terms associated with specialized nursing, patient safety, and telehealth. To cover most of the peer-reviewed and scientific international literature on the review phenomenon, key online databases such as PubMed (including MEDLINE), Scopus, CINAHL, Web of Science, ProQuest, and Embase were selected. Additionally, a librarian was consulted to ensure the accuracy of the search process.

### Selecting relevant studies

For inclusion in this review, studies should have focused on telehealth and navigated by specialized nurses in short-term, long-term, or community care settings for adults living in their own home receiving physical and mental healthcare. The studies should have recognized the expanded responsibilities assigned to nurses, justifying their classification as specialized professionals, and should have clearly outlined their roles, responsibilities, and perspectives within telehealth initiatives. Original and empirical studies utilizing qualitative, quantitative, or mixed methods, and being published in scientific peer-reviewed journals in English were considered. The publication date was restricted to the last decade, from January 1, 2013, to August 29, 2024.

Commentaries, letters, case reports, simulation studies that did not involve real patients in clinical practice, case studies and books that lacked empirical data or did not align with the primary domains of this review were excluded. Studies centered on pediatrics, child, and neonatal care were excluded due to the profound differences in clinical considerations that distinguished specialized nursing practices for them from adult care. This ensured a concentrated exploration of the unique challenges, treatment modalities, and outcomes associated specifically with adult care. To enhance the search coverage, a manual search was also conducted within reputable journals known for publishing studies in the fields of advanced nursing practice and digital health. Moreover, cross-referencing from the bibliographies of selected studies was also employed to ensure a comprehensive search.

### Charting, collating, and summarizing data

The search results were uploaded to Rayyan.ai, serving as an online platform conducive to teamwork in performing review studies. It facilitated a systematic screening and selection process adhering to predetermined eligibility criteria applied to studies’ titles, abstracts, and full texts independently by two review authors (MV, PAL). Through shared online discussions, they collaboratively decided on the review process, resolving disagreements through further discussions to reach a consensus on the inclusion or exclusion of studies.

While quality appraisal in scoping reviews may not be conventionally applicable, the chosen studies were subjected to assessments to ascertain if they possessed necessary methodological standards and provided reliable insights into the review phenomenon. Customized JBI Critical Appraisal Tools^39^ tailored to the distinct research methodologies encompassing cohort, experimental, and qualitative approaches were employed. Each study was evaluated by the review authors independently, which guided the decision-making process of whether to include or exclude studies in the research synthesis, considering their methodological rigor.

### Reporting results

The review authors screened the titles and abstracts of the selected studies against predefined eligibility criteria, proceeding to the independent examination of their full texts. Consensus on article selection and inclusion in reporting was reached through collaborative discussions and shared findings. Data extraction involved systematic organization in a table for easy comparison and categorization of both general and specific characteristics relevant to the review focus. Collaborative teamwork integrated study findings through a comparative analysis of specialized nurses’ roles in using telehealth for patient safety, resulting in the development of meaningful categories. This review followed the Preferred Reporting Items for Systematic reviews and Meta-Analyses (PRISMA)^40^ checklist and its extension for Scoping Reviews (PRISMA-ScR) for its development and reporting^41^ (Supplementary File 3).

### Seeking consultation

The consultation step, usually considered optional, aims to engage stakeholders for additional references and insights beyond the reviewed literature. However, due to constraints like ethical permissions and funding, the authors excluded this step. Nonetheless, the inclusion of qualitative studies in the research synthesis aimed to address potential limitations from this omission. Qualitative research findings helped enhance our understanding derived from quantitative studies as they often provided contextual insights into the experiences of incorporating telehealth into nursing practice and offered a deeper comprehension of barriers and facilitators influencing telehealth implementation.

## Results

### Search outcome and selection of studies

**Figure 1.**
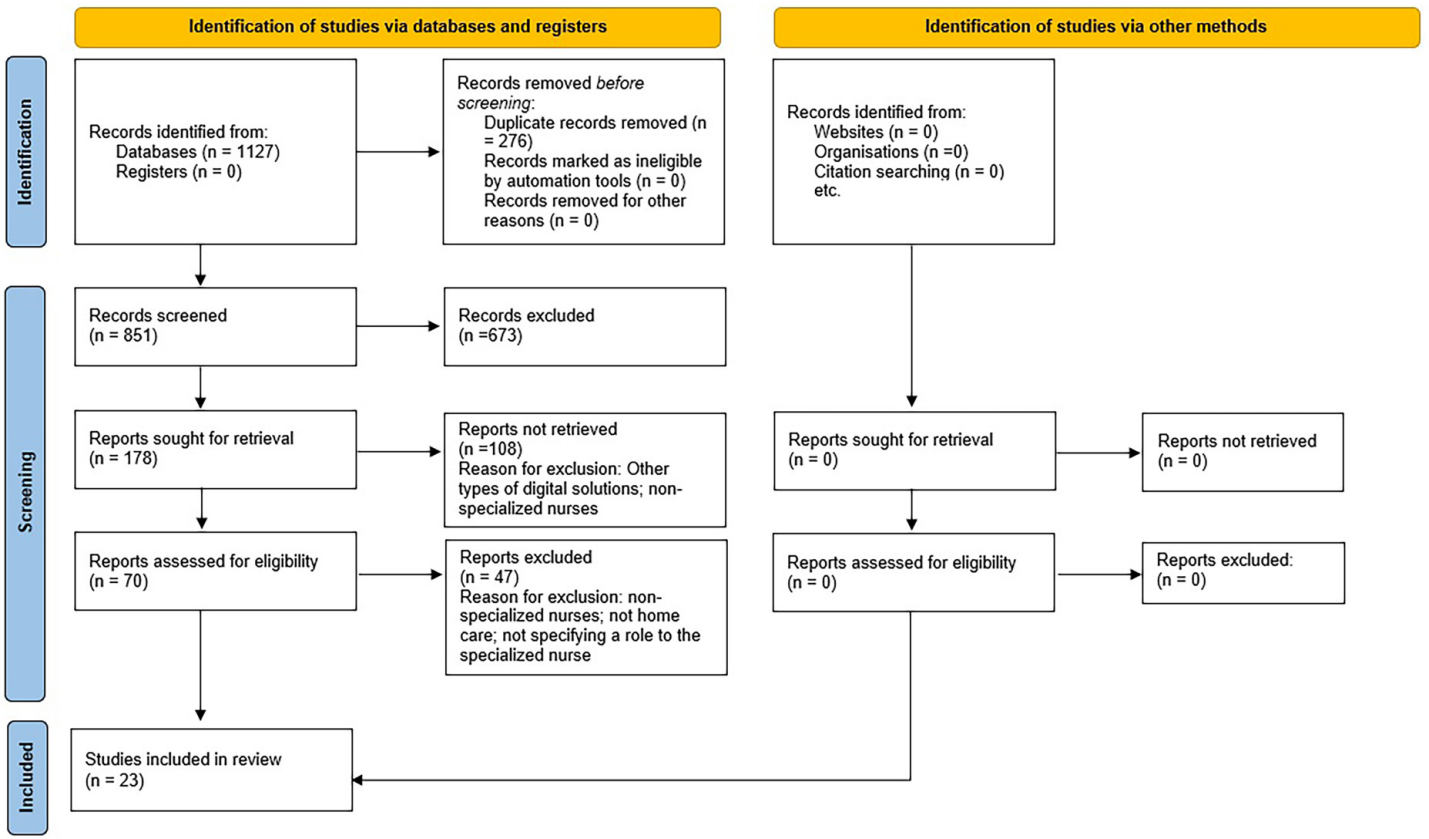
The search process.

The comprehensive database search identified 1127 studies (Table 1). After removing duplicates, eliminating irrelevant studies such as other types of digital solutions and non-specialized nurses, the final selection narrowed down to 23 studies for research synthesis. The search process is visually outlined following the PRISMA guideline in Figure 1.

**Table 1.** The search results.

Databases from 2013 to 2024 (last decade)	Total in Each Database	Screening of Title and Abstract	Full-Text Reading and Quality Appraisal
PubMed (including MEDLINE)	59	16	2
Scopus	171	29	5
CINAHL	310	74	10
Web of Science	346	41	5
ProQuest	232	18	1
Embase	9	0	0
Manual search/backtracking references	–	–	0
Total of databases	1127	178	23
Duplications	276	0	0

### Quality appraisal

Experimental studies,^42–60^ employed suitable methods for sampling, recruitment, and outcome measurements. The cross-sectional study^61^ and the cohort study^62^ provided detailed descriptions of sampling and recruitment processes, along with comprehensive information on exposure and its measurement. In the qualitative study,^63,64^ the research design demonstrated transparency in data collection and analysis, ethical considerations, and the credibility of findings. Consequently, all selected studies were deemed to have sufficient quality, justifying their inclusion in the data analysis and research synthesis (Supplementary File 4).

### General characteristics of the selected studies

The selected studies, published in English between 2014 and 2024, originated from the USA,^42,44,45,48,52,56,61,62^ the Netherlands,^43^ the Netherlands-Belgium,^58^ Canada,^46^ Australia,^47,49,53^ Ireland,^50^ New Zealand,^51^ Germany,^54^ the UK,^55,63,64^ Finland,^57^ China,^60^ and Iran.^59^ In total, the studies included 13,190 participants undergoing telehealth interventions. Other characteristics of the studies are summarized concisely in Table 2.

**Table 2.** Characteristics of the studies selected for research synthesis

Author, y	Country	Design/Focus	Sample	Research Outcome	Specialized Nurse and Description of Navigation Role via Telehealth	Type and Method of Navigation via Telehealth
Goldman et al. (2014)^45^	USA	Randomized controlled trial/ Nurse-led pre-discharged intervention in comparison with usual care as bedside discharge; nurses and NPs received support from the healthcare team	*n* = 700 older adults in a single acute-care hospital	Visits to ED and readmissions within 6 months as the percentage of patients’ visit (2–5 times) to the ED	NP: giving education about medication side effects and warning signs to sensitize patents and proactively seek care	Telephone follow-up for symptoms, adherence to medications and treatment plan on days 1–3 and 6–10 after discharge to home
Vuorinen et al. (2014)^57^	Finland	Randomized controlled trial/ Self-assessment and report against multidisciplinary care standard practice	*n* = 47 patients with HF at a cardiology outpatient clinic and *n* = 47 as control	Hospitalization days during the follow-up, clinical outcomes, unplanned visits, individual acceptance/experience, self-care behavior within 6 months	Specialized HF nurse: providing up-to-date information about patients to support physicians for decision-making about medication management; monitoring medications’ taking precisely by patients and encouraging self-monitoring	Telemonitoring via mobile phone app on a weekly basis or as required contacted patients at home, and supporting self-assessment
Brainerd and Hawkins(2016)^42^	USA	Evidence-based practice project/Supporting patients with HF by primary healthcare provider and HF registered nurse	*n* = 6 patients with HF	Enrolment with the program and reduction in the number of 30-day hospitalization within 9 months	Advanced practice registered NP: making decision for patient management in terms of education, making medication adjustments, and planning for referrals to emergency settings	Home telehealth on an individual's need basis for education via telephone and electronic device in veterans’ home for monitoring daily weight, BP, HR, and blood glucose
Ishani et al. (2016)^48^	USA	Randomized clinical trial/Care delivery affecting health outcomes using interprofessional team *vs.* usual care	*n* = 451 patients with chronic kidney disease in the intervention and *n* = 150 in the control group at a veterans’ healthcare system and affiliated community-based clinics	Death, hospitalization, ED visits, admission to nursing care home within 12 months	NP: providing support for developing individual-specific care plan including medication management, symptoms management, and life-style modifications	Telehealth device for in-home visit based on the patient's need through a video monitoring device with peripherals and broadband
Moore (2016)^52^	USA	Pilot intervention/Nurse-led interdisciplinary healthcare program in home care for care management, early assessment, and intervention	*n* = 22 patients with chronic HF at a Medicare certified home health agency serving two northeastern states	Exacerbation of cardiac symptoms and 30-day readmission within 4 months	NP: performing early and timely home assessments via phone follow up, education, daily telemonitoring, symptoms’ self-management, early detection of care issues, changing medications’ doses, and planning for prevention of unnecessary readmissions	Telemonitoring and weekly telephone home follow up weekly for 9 weeks for reporting data about BP, HR, weight
De Heide et al. (2017)^43^	Netherlands	A single center pilot study/Wound assessment	*n* = 46 patients undergoing elective cardiac interventions at a clinical electrophysiology department	Ability to upload wound picture, image interpretability, agreement, and patient evaluation of the intervention within 3 months	NP: evaluating wound pictures for clinical significance, care planning, and managing conservative therapy	Telemedical device for wound assessment as a mobile phone for contact at day 7 after discharge to home
Fisher et al. (2019) ^62^	USA	A prospective pilot cohort/Developing a remote home-based control program for blood pressure managed by NP and pharmacists	*n* = 130 patients with hypertension at a primary care practice and a principal cardiology clinic	Rapid medication titration and ensuring the program efficacy and safety within 6 months	NP: performing medication titrations in the medication management team every two weeks based on an expert-developed clinical algorithm as rapid assessment-treatment cycles and training of patient in the process	Remote, navigator-led hypertension innovation program leveraging algorithmic care pathways home for blood pressure with measurements transmitted wirelessly into the electronic medical record for medication titration by phone at biweekly intervals
McNeal et al. (2019)^61^	USA	Descriptive cross-sectional/Addressing the healthcare needs of underserved patients by the interprofessional primary healthcare team: nurse, social worker, and physician	*n* = 477 underserved inner-city residents at three communities	Loss to follow-up and cost effectiveness within 3 years	Advanced practice nurse: providing direct care services with the utilization of telehealthcare for medical treatment, scheduling follow up care and referral for specialty care	Telehealthcare at home as virtual nurse-managed clinic based on patients’ needs in terms of cloud computing, teleconferencing, and biometric devices over compliant remote networks
McGloin et al. (2020)^50^	Ireland	An observational, pre-post, multimethod, and triangulation/Clinical effectiveness, and feasibility of a supportive intervention by specialized nurse and community care clinic	*n* = 39 patients undergoing insulin therapy from hospitals and community-based diabetes clinics	Patient empowerment and diabetic control within 3 months	Diabetes clinical nurse specialist: evaluating medication use, and making necessary adjustments and lifestyle changes	Home telemonitoring via telephone and a hub for blood glucose recording and insulin therapy based on the patient's need
Ruf et al. (2020)^55^	UK	Intervention/Distance patient care management	*n* = 88 patients with inflammatory bowel disease living in remote rural areas	Patient safety and economic benefit for patients within 15 months	Specialist nurse: assessing location/behaviour of disease, defining disease severity, and planning for referral	Videoconference clinics based on patients’ needs as a distance-management tool at home
Seuren et al. (2020)^63^	UK	Qualitative microanalysis of video examinations using conversation analysis/Challenges with remote physical examination of patients	*n* = 7 patients with HF in a community	Physical examination	Community-based specialist nurse: making judgment of the adequacy and trustworthiness of clinical examinations	Video consultations at home using facetime as pilot testing
de Peralta et al. (2021)^44^	USA	Intervention/Virtual care to patients based on a collaborative model during COVID-19 pandemic	*n* = 132 patients as veterans with HF	Face to face care and referral to emergency department within 3 months	NP: assessing patients’ vital signs and symptoms, and supervision of care	Telemedicine via phone or video-to-home on a weekly basis
Ho et al. (2021)^46^	Canada	A randomized controlled trial/Feasibility of remote monitoring home care	*n* = 70 patients with HF from three urban hospitals	Unscheduled ED revisits within 90 days, all-cause death, hospital readmission, quality of life, self-efficacy, end-user experience, and cost-effectiveness	Cardiac specialty nurse: monitoring data related to symptoms for customizing patients’ needs leading to advice, provision of education to patients and getting support from physician, and planning for referral	Home health monitoring solution to monitor weight, BP, HR, and to answer symptomology questions via Bluetooth sensors and tablet computer on a daily basis
Indraratna et al. (2021)^47^	Australia	Mixed-methods process evaluation of a pilot 2-centere randomized controlled trial/A novel model of monitoring care by a team of specialized nurse and cardiologist	*n* = 83 patients with acute coronary syndrome and HF in the intervention and n = 64 as control from two hospitals	Cardiac rehabilitation completion, referrals, and cardiologist follow-up appointments within a 6-month period	Cardiology NP: monitoring vital signs and symptoms, educating patients, and performing home visitation	Telemonitoring based on the patient's needs as a smartphone app for monitoring BP, weight, HR reading at home
McLachlan et al. (2021)^51^	New Zealand	Feasibility pilot intervention/Remote monitoring of vital signs and symptoms during COVID-19 pandemic with support of cardiologist if needed	*n* = 50 patients with HF	Number and time of contacts, time to maximal tolerated therapy, reason for variation in titration, hospitalization, death, change in clinical parameters within 4 months	NP: monitoring medications for titrating	Telephone home support every two weeks for fluid congestion, monitoring vital signs
Maher et al. (2022)^49^	Australia	Pilot intervention/Efficacy of a nurse-led model with support of gastroenterologist if needed	n = 134 patients with positive fecal occult blood test referred to a public tertiary center and *n* = 49 standard outpatient clinic	Time to colonoscopy, adverse events, bowel preparation, cancellation rates within 14 months	Clinical nurse consultant: providing education and screening of patients, and making decision for colonoscopy and related preparations	Virtual clinic as appointments on electronic booking system and automatic generation of short message reminders based on the patient's needs at home
Nehme et al. (2023)^53^	Australia	Prospective study/Access to treatment, saving healthcare, resources and cost-efficiency	*n* = 9588 patients with non-urgent mental health-related complaint in an urban area	Proportion of video telehealth cases and referrals to an alternative health service, cost effectiveness within 15 months	Mental health nurse specialist: providing self-care advice and referral	Home video telehealth as a video streaming between the caller and the nurse using a mobile phone based on the patient's needs
Prescher et al. (2023)^54^	Germany	Randomized trial/Remote patient management by a nurse specialist with advanced training in HF management	*n* = 765 patients with HF in the intervention group and *n* = 773 usual care group from 200 university, local, and regional hospitals, and cardiology and general practitioner practices	Experiences and adherence to daily measurement within about 5 years	HF nurse specialist: assessing disease status, managing emergencies, changing treatments, and verifying measurements	Telemedical home intervention as app on a tablet to measure and transmit automatically daily vital signs, and telephone call on a monthly basis
Schmaderer et al. (2023)^56^	USA	Pilot randomized trial/Self-management and self-monitoring at home post discharge by NPs as specialist in cardiology	*n* = 74 patients with HF from a hospital: *n* = 26 in the enhanced usual care group, *n* = 23 in mHealth group, *n* = 25 in mHealth plus group	Adherence to medications and weight monitoring within 3 months	NP: developing self-management strategies and action care plans	Mobile home health intervention as an app with a Bluetooth-enabled scale to record prescribed medications at scheduled times and weights, and hold virtual visits at weeks 2, 5, and 8 after discharge
Zhang and Guo (2023)^60^	China	Intervention/Internet-based telehealth nursing	*n* = 168 patients with atrial fibrillation and stroke: 84 in the internet-based telehealth nursing group and 84 in the traditional at-home self-help nursing group	Survival rate, readmission rate, daily activities, limb motor ability, psychological state, cardiac function, rhythm control, adverse events, and nursing satisfaction	3 nurse stroke specialists in cardiovascular medicine and 3 stroke nurse specialists in neurology: arranging audio and video calls, guiding patients and families, following up, providing individualized care	Audio and video calls, WeChat video, wearable ECG, home BP monitoring device on a weekly basis for 1 year
Anderson et al. (2024)^64^	UK	Qualitative/Nurses in general practice experienced remote and technology-based work during the COVID-19 pandemic	8 ANPs	Issues of technology access, workload, hybrid work, disrupted relationships, safety risks, decision-making exclusion	ANP: video consultations and telephone follow up of patients in home care	Telephone and video call: sending care plans and care techniques videos, clinical information, consultation, in-person care
Leenen et al. (2024)^58^	Netherlands/Belgium	Prospective, international, multicentre, single-arm interventional study/Perceived usability of a digital platform in addition to hospital at home	63 Patients with acute decompensation of pre-existing HF	Perceived acceptability, appropriateness, feasibility, and satisfaction of healthcare providers within a 15-month period	Specialist HF nurses as 18 cardiac care nurse and 1 NP: following up patients remotely, sending individual alerts to optimize monitoring	A digital health platform consisting of portable BP, weight scale, pulse oximeter, a wearable chest patch for HR, respiratory rate, activity level, an eCoach on a mobile application to receive measurement reminders and track evolutions
Mirshahi et al. (2024)^59^	Iran	Single-site, pilot randomized controlled trial/Feasibility and acceptability of a telehealth palliative care intervention	50 patients with HF: 25 in each intervention and control group	Feasibility, acceptability, quality of life, satisfaction, attrition, anxiety, depression, visit to the ED within 12 weeks	1 nurse interventionist: leading a comprehensive 6-week virtual program as weekly webinars, sharing case scenarios in groups and encouraging patients to discuss and comment	WhatsApp and SkyRoom for implementing the educational program as webinars, questions and answers meeting, working with case scenarios, support in emergencies and addressing concerns

*Note*. ANP: advanced nurse practitioner; NP: nurse practitioner; BP: blood pressure; COVID-19: coronavirus disease 2019; ECG: electrocardiography; ED: emergency department; HF: heart failure; HR: heart rate.

### Specialized nurses’ roles in telehealth

**Figure 2.**
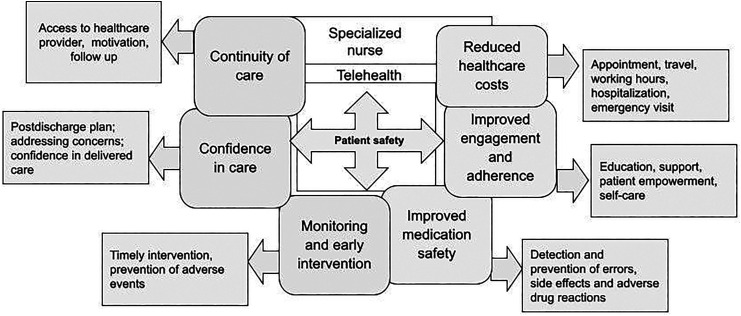
Utilization of telehealth by specialized nurses in relation to patient safety in home care.

Specialized nurses played a crucial role in harnessing telehealth within the home care context in connection with patient safety. Their responsibilities as remote navigators included facilitating virtual appointments with patients,^60,61^ remotely managing and monitoring their health records,^43,44,46–48,50–52,54,57,58,64^ education and training,^42,45–49,52,53,56,57,59,60,62,64^ referral support,^42,46,52,53,55,61^ and decision-making and assistance for decision-making.^42,46,49–51,54,56,57,62,63^ Therefore, they ensured patients’ seamless access to healthcare providers and healthcare systems as well as empowered them to manage their health at home. The practical descriptions of the specialized nurses’ role as telehealth navigators are outlined in Table 2. The review results were presented based on key outcomes linked to the use of telehealth by specialized nurses to preserve patient safety in home care in Figure 2.

### Scope of specialized nurses’ practice in telehealth

#### Continuity of care

As a patient-centered healthcare experience, telehealth use by specialized nurses empowered patients to proactively manage their health through consistent communication and follow-up with the healthcare team and adhere to prescribed therapeutic regimens.^44,48,54,57,61^ However, one study found that specialized nurses reported telehealth could jeopardize therapeutic relationships, heighten safety risks due to patients’ difficulties with clinical procedures, and raise privacy concerns, making thorough patient assessment more challenging.^64^ This perspective was also noted in a study on the use of health platforms to support home hospitalization for heart failure patients in the Netherlands and Belgium, where a subset of patients struggled with self-management in performing the required measurements.^58^

The implementation of the phone or video-to-home solution for patients in the USA guided by an algorithm for patients dealing with heart failure resulted in a 100% (*n* = 132 visits) success rate in transitioning face-to-face appointments to telemedicine.^44^ Over a span of 3 years, care delivery via the virtual nurse-managed clinic in the USA effectively administered the treatment for 477 underserved inner-city residents suffering from diverse physical and psychological ailments, totaling 1561 visits during this timeframe.^61^ Similarly, during in-home visit through a video monitoring device in the USA for patients with the chronic kidney disease, a total of 850 vital signs triggered kidney disease-specific flags, requiring contact. The median was one contact per veteran, while the maximum reached 19 contacts per person.^48^ In the Netherlands and Belgium, the digital platform was used 79% of the time during home hospitalization days for heart failure patients, with an overall usability score of 72.1 ± 8.9.^58^

As for monitoring healthcare, a telemedical intervention via an app designed for patients with heart failure in Germany demonstrated an adherence rate of over 85% (*n* = 670) among the participant population.^54^ Patients with heart failure receiving telemonitoring via app in home care in Finland did not exhibit any change in the number of hospitalization days (incidence rate ratio (IRR) = 0.812) compared to the multidisciplinary standard practice. However, an elevated frequency of visits to the nurse (IRR = 1.73), extended time spent at the nurse reception (mean = 48.7 min), and a higher number of telephone contacts between the nurse and intervention patients, both initiated by the nurse (IRR = 3.82) and also in response to patient-initiated contacts (IRR = 1.63) were observed.^57^

#### Confidence in care

Specialized nurses using telehealth alleviated stress among patients and their family caregivers during the post-discharge trajectory of care, leading to overall satisfaction and feeling of safety with care and confidence in the healthcare system.^44,46,49–51,53,54,56,63^

Patients undergoing insulin therapy via telephone monitoring in Ireland reported highlighted awareness and skills for self-managing diabetes, increased feeling of safety and comfort, as well as a rise in empowerment and responsibility for their conditions, and reduction in distress. They attributed these positive changes to the assurance that someone consistently monitored their blood glucose levels. This continuous oversight, coupled with enhanced patient self-management skills resulting from increased knowledge and confidence, contributed to an overall improvement in the patient experience. The mean patient satisfaction with the intervention consistently scored above 4 (maximum 5) on all items indicating feeling trust and safety in care delivered by this method.^50^ For veterans with heart failure receiving the phone or video-to-home intervention in the USA, this platform served as a satisfactory medium for the delivery of consultations. Moreover, it proved to be a dependable tool for identifying patients who required emergency or hospital-level care, contributing to the effectiveness of remote healthcare management for this specific patient population.^44^ Among Iranian heart failure patients receiving telehealth palliative care, satisfaction with webinar sessions had a mean score of 3.71 ± 0.94, with 64.4% scoring ≥4. Satisfaction with WhatsApp group activities had a mean score of 4.02 ± 0.81, with 73.3% scoring ≥4.^59^ Internet-based telehealth nursing for atrial fibrillation and stroke patients in China led to higher patient satisfaction than the control group.^60^

Patients with heart failure receiving telephone support during COVID-19 pandemic in New Zealand reported that the process was deemed acceptable. They underwent rapid medication titration leading to a reduced necessity for clinic reviews. Participants showed confidence with the use of blood monitoring devices at home, as they felt engaged and empowered to participate in their own care.^51^

A home health monitoring for patients with heart failure in Canada via a tablet yielded positive outcomes, notably enhancing their self-efficacy. Patients expressed overall satisfaction with the monitoring program, citing its usability. Patients expressed overall satisfaction with the monitoring program, citing its usability. Moreover, on a 100-point scale, the mean score of 80.0 (median of 81.4) indicated participants felt more engaged, educated, and involved in their self-management. This underscores the effectiveness of the monitoring solution in not only improving patient satisfaction but also promoting active participation in self-care.^46^

Patients with heart failure (98.9%, *n* = 559) undergoing an intervention via an app in Germany expressed satisfaction with both the telemedical intervention program and the usability of the associated devices. Also, 81% (*n* = 361) of these patients expressed a desire to, or could envision, continuing their care through telemedicine, highlighting the positive reception and potential for ongoing adoption of telemedical interventions in managing heart failure.^54^ In the USA, most patients (94%, *n* = 34) with heart failure undergoing the health intervention via a mobile app were satisfied and found it to be feasible, usable, and acceptable. Also, 93% (*n* = 33) of them concurred that they learned how to effectively self-manage their heart failure. Furthermore, 83.3% (*n* = 34), expressed satisfaction with the amount of information received about heart failure during virtual visits. Notably, 88.9% (*n* = 33) of patients affirmed that the virtual visits contributed to an increased sense of confidence in managing their health and the entire participant group acknowledged the value of the intervention in enhancing their ability to self-manage their heart condition.^56^ Healthcare professionals including specialized nurses in the Netherlands and Belgium felt confident using the digital platform to support home hospitalization for heart failure patients (*n* = 20; 60.6%). The platform gave them better insight into clinical trends and helped prepare for home visits, enhancing the feeling of safety for patients at home.^58^

The individuals (26 out of 30) awaiting colonoscopy overwhelmingly endorsed to others the virtual clinic service and reminders in Australia, highlighting an overall positive experience with undergoing the procedure without the need to physically attend the gastroenterology clinic implying confidence in the approach used.^49^ An impressive 92.8% (*n* = 120) of patients in Australia with non-urgent mental health-related concerns expressed satisfaction with the video streaming service via mobile phone in the follow up, indicating a favorable view of its efficacy. Additionally, 89.4% (*n* = 115) of patients conveyed their intention to utilize video telehealth again if they were to seek assistance in the future.^53^ For patients with heart failure in the UK, the use of both verbal descriptions and visual depictions was a common practice within the video consultation intervention and indicated instrumental in providing instructions through the video medium to patients. Patients appreciated video examinations as an opportunity to acquire knowledge on self-assessment and develop skills to manage their own medical condition.^63^

#### Monitoring and early intervention

Telehealth facilitated an early identification of potential health issues by specialized nurses, enabling timely interventions, and preventing readmissions, unnecessary visits, and negative health consequences.^42,44–47,49–53,55,57^

As evidence for the use of telephone and electronic device for patients with heart failure in the USA, it is noteworthy that 50% (*n* = 3) of individuals experienced readmissions before the implementation of the project. In contrast, none of the patients (0 out of 24) enrolled in the project were readmitted to the hospital, highlighting a significant effect of the practice change. This outcome, specifically the absence of admissions, has collectively extended the interval between hospital readmissions for the entire cohort, as 50% (*n* = 3) of pre-project patients had readmissions, but none of the intervention patients were readmitted, underscoring the positive influence of the telehealth initiative.^42^ In telemedicine as video-to-home in the USA, only three veterans with heart failure were referred for further care to the emergency department.^44^ The telemonitoring program based on weekly telephone follow ups designed for patients with chronic heart failure in the USA demonstrated its effectiveness by reducing 30-day readmission rates to 9% (*n* = 2), a notable improvement compared to the national average of 23%. This initiative resulted in a substantial decrease in 30-day hospital readmissions and emergency department visits through changes in medications’ doses such as Lasix and antibiotics, particularly within one home health agency providing home health services. The all-cause readmission rate for this specific group dropped significantly to 9%, showcasing a remarkable reduction from the initial rate of 27.8% (five out of seven readmissions).^52^ For heart failure patients in New Zealand, the home monitoring process via telephone support resulted in rapid medication titration and less need for clinic reviews and 60% (*n* = 129) of clinic visits were held remotely saving nurses and patients time.^51^ Telemonitoring via telephone for patients undergoing insulin therapy in Ireland was translated to a more rapid intervention, consequently diminishing the necessity for hospital visits as only 7% (*n* = 3.39) of patients experienced unplanned hospital admissions.^50^ A study on internet-based telehealth nursing for patients with atrial fibrillation and stroke in China reported an 84.5% 1-year survival rate, with significantly fewer readmissions due to stroke and complications compared to the control group.^60^

Providing education to older adults in the USA through telephone follow-ups in comparison with bedside discharge regarding medication side effects and important warning signs did not necessarily reduce the hazard ratio of emergency department visits (intervention vs. control: 1.26 (95% CI: 0.89–1.78) at 30 days, 1.21 (CI: 0.91–1.62) at 90 days, and 1.11 (CI: 0.86–1.43) at 180 days). Nevertheless, it had a sensitizing effect, resulting in a proactive approach to seeking care in advance, even for situations that would not pose any harm.^45^ The implementation of video streaming via mobile phone for emergency secondary triage in patients with mental health issues in Australia was correlated with a reduction in emergency ambulance dispatches and an increase in referrals to alternative services. It was associated with reduced odds of emergency ambulance dispatch (OR = 1.009) and increased referrals to alternative services (OR = 1.321) compared to voice only.^53^

The videoconference clinics for patients with inflammatory bowel disease in the UK demonstrated both safety and effectiveness, with only 0.9% (*n* = 2) of appointments necessitating urgent medical assessment. A substantial 92% (*n* = 211) of appointments simply required further virtual follow-up, underscoring the overall success in monitoring and follow up of these patients. In 7% (*n* = 16) of appointments, there was a need for referral to another department or allied health professional.^55^

Internet-based telehealth nursing for atrial fibrillation and stroke patients in China reduced the incidence of complications, including deep vein thrombosis, hemorrhage, pulmonary infections, urinary infection, aspirations, and fall-related trauma compared to the control group.^60^ Home health monitoring via sensors and the tablet caused a significant reduction across various healthcare metrics for patients with heart failure in Canada. This included a remarkable 79% decrease in emergency department revisits, an 87% reduction in hospital readmissions, and a substantial 60% decrease in the median hospital length of stay.^46^ Two thirds (66%, *n* = 29/44) of patients with heart failure receiving mobile phone app for self-assessment in Finland reported that the feedback they received from specialized nurses assisted them in focusing more on crucial aspects of their treatment. It served as a motivating factor for consistently taking measurements and reporting them, ultimately prompting positive changes in their lifestyle.^57^

In contrast to the traditional outpatient clinic model, the virtual care model on the electronic booking system and reminders for patients at high risk of colon cancer in Australia significantly decreased waiting times for colonoscopy, reducing the duration by 71 days from the date of referral and by 66 days overall. Additionally, this virtual care model was associated with a reduction in the risk of adverse events as the nurse efficiently scheduled appointments, coordinated timely colonoscopies, diligently followed up on results, and effectively communicated them with patients.^49^ The simplification of the enrollment process through telemonitoring via an app improved the screening of cardiac patients in Australia, leading to a 50% (*n* = 21, hazard ratio = 0.51, 95% CI, 0.31–0.88) reduction in total readmissions compared to the standard care group within 6 months.^47^

#### Medication safety

Telehealth operationalized medication management by specialized nurses through safe medication administration and prescription based on early reports from patients and diligently tracking signs and symptoms to identify and address potential side effects and adverse drug reactions.^50,51,57,62^

In telemonitoring for heart failure patients in Finland, participants adhered precisely to their medication regimens and experienced a higher frequency of medication adjustments, including both reductions and increases in medication usage, which involved modifications to diuretics, angiotensin-converting enzyme inhibitors, and beta-blockers.^57^ A hypertension management program, guided by remote navigators, successfully achieved blood pressure control without significantly escalating the pill burden. Using the remote algorithmic care pathways for blood pressure among patients with hypertension in the USA, the average number of medications used rose modestly from 1.4 to 1.8 during the transition from baseline to control, with Amlodipine being the most frequently added new medication.^62^

Telephone support for heart failure patients in New Zealand resulted in optimal titration for 75% (*n* = 38) of individuals within a span of 2 months. Notably, a significant milestone in medication regimen was achieved with 88% (*n* = 44) reaching or surpassing 50% of the target dose for the renin-angiotensin blocker, 74% (*n* = 37) achieving the same for the beta blocker, and 62% successfully incorporating spironolactone into their medication regimen.^51^

Telemonitoring for insulin therapy involved vigilant monitoring of medication therapy and offered support to symptomatic patients (*n* = 15, 38%) in Ireland. This proactive approach resulted in discontinuation or dose adjustment, primarily in response to incidents of hypoglycemia. The most common intervention was the adjustment of insulin dosage, implemented on one or more occasions, followed by the provision of advice on healthy eating and physical activity.^50^

#### Engagement and adherence

Telehealth assisted specialized nurses in educating, engaging, and empowering patients, which in turn, enhanced patients’ adherence to treatment plans, facilitated successful care outcomes and thereby decreasing the likelihood of adverse events.^43,46–48,50,51,53,54,56,57,62^

A notable 83% (*n* = 33) of patients receiving telemedical interventions for wound assessment in the Netherlands were able to participate in their own care and uploaded pictures of their wounds, with the majority being easily interpretable Only 8% (*n* = 3) of patients undergoing elective cardiac intervention expressed a preference for a hospital visit indicating their engagement with the current program.^43^ Similarly, 78% of heart failure patients in Iran receiving telehealth palliative care from specialized nurses said they would recommend the service to others, with an average adherence score of 7 ± 1.02.^59^

Heart failure patients undergoing home health monitoring in Canada demonstrated a high level of engagement, with an impressive adherence rate reported at 94% (*n* = 70) for the monitoring protocols.^46^ Video telemonitoring in Australia enhanced the assessment of mental health issues by incorporating visual cues, thereby improving patients’ engagement in the process.^53^ Telemonitoring in cardiac patients in Australia enhanced their satisfaction from 48% (*n* = 34 out of 71) to 75% (*n* = 56 out of 76), which positively impacted self-care practices, and adherence to cardiac rehabilitation (OR = 2.9) and prescribed medications.^47^

Although mortality (hazard ratio (HR) = 1.46, 95% CI: 0.42–5.11), hospitalization (HR = 1.15, CI: 0.80–1.63) and emergency department visits (HR = 0.92, CI: 0.68–1.24) did not differ between the interprofessional team telehealth and routine care in the USA, patients with chronic kidney diseases who were part of telehealth actively participated in the intervention through multiple virtual visits. Remarkably, 96.2% (*n* = 434) of them successfully completed at least one video visit as part of the intervention.^48^

The provision of telephone support for patients with heart failure in New Zealand caused notable improvements in key health indicators. Systolic blood pressure decreased from 124 to 116 mmHg, pulse decreased from 78 to 70 bpm, and N-terminal pro-brain natriuretic peptide levels decreased from 292 to 65. Additionally, a substantial 77% (*n* = 33) of patients exhibited significant improvement in left ventricular ejection fraction. Participants experienced enhanced titration rates and improvements in cardiac imaging, biochemical parameters, and clinical findings. Patients’ improved comprehension and willingness to accept alterations to their medication regimen contributed to program success.^51^

In the telemedical intervention for heart failure patients in Germany, a robust and consistently high level of adherence, measured at 89.1% ± 14.1% (*n* = 682) throughout the study duration was observed. This adherence remained consistently high regardless of the disease severity. Notably, patients residing in rural areas exhibited higher levels of complete adherence compared to those in urban regions.^54^ There was an observable upward trend in the proportions of patients with heart failure utilizing an app for recording medications in the USA, with more than 50% (*n* = 13) consistently engaging in this activity. Furthermore, there was a noticeable trend, also surpassing 50%, indicating improved adherence to weight-related measures.^56^

Heart failure patients in the telemonitoring group in Finland regularly self-monitored their body weight, blood pressure, and pulse. Additionally, they responded to symptom-related questions on a weekly basis, with their recorded values submitted accordingly. The adherence rate for this self-monitoring process was reported to be about 90%.^57^

In terms of outcomes, 91% (*n* = 105) of patients who consistently monitored their home blood pressure within the remote navigator-led program in the USA successfully reached their goals, achieving this milestone in an average of 7 weeks. The systolic blood pressure in these patients decreased from the baseline clinic pressure of 155 ± 18 mm Hg to 124 ±  8 mm Hg, and the diastolic blood pressure dropped from 92 ± 13 mm Hg to 74 ± 8 mm Hg.^62^

For patients receiving telemonitoring for insulin therapy in Ireland, no change in weight was observed, but the mean hemoglobin A1c (HbA1c) decreased with a noticeable clinical significance (mean difference: 17.3, 18.16).^50^

#### Reduced healthcare costs

Telehealth increased specialized nurses’ workload as algorithm-based systems where patients submitted online forms, added to their tasks during the COVID-19 pandemic.^64^ Also, in the Netherlands and Belgium, 73% of healthcare providers including specialized nurses reported that the digital health platform used to support home hospitalization for heart failure patients led to an increased workload.^58^ Nevertheless, it reduced healthcare cost concerns, ultimately enhancing patients’ quality of life, and improving adherence to the telehealth program.^46,51,53,55,61^

Telemonitoring of patients with heart failure in home care in Canada resulted in a 71% decrease in hospitalization costs and a 58% (*n* = 30) reduction in emergency department visit expenses. Additionally, there was an overall 56% (*n* = 30) decrease in health system costs for patients, 71% reduction in hospitalization costs, coupled with a notable 100% improvement in the quality of life related to heart failure.^46^ Among Iranian heart failure patients, improvements in quality of life were significant compared to the control group, with mean differences of −1.54 (95% CI: −2.14 to −0.94) and −2.69 (95% CI: −4.22 to −1.16).^59^ A study on internet-based telehealth nursing for patients with atrial fibrillation and stroke in China significantly reduced mean anxiety (33.1 ± 8.0) and depression (33.8 ± 3.3) levels at 1-year follow-up.^60^

Telephone support for patients with heart failure in New Zealand removed travelling costs averaging $NZ58.17 per patient.^51^ The virtual nurse clinic for inner-city residents in the USA proved to be cost-effective, with a cost savings of $US10 for every dollar spent on a visit, specifically contributing to the prevention of emergency room visits that could have been avoided.^61^ Video triage for mental health patients in Australia incurred an average cost that was half of voice triage, amounting to $A970.8, which in turn, was half the cost of a conventional secondary triage.^53^

The implementation of a videoconference clinic for patients with bowel disease in the UK resulted in a savings of $US36.61 per appointment, representing the potential travel cost that would have otherwise been incurred. Also, patients of working age experienced a total savings of 1037.3 lost work hours in travel time throughout the study period. On average, potential savings of $36.61 in traveling costs per appointment could be realized.^55^

## Discussion

This review aimed to identify the nature and scope of specialized nurses’ roles in ensuring patient safety within the context of telehealth in home care. The review results showed that specialized nurses improved patient safety by leveraging telehealth for patients in home care through continuity of care, confidence in care, monitoring and early intervention, improved medication safety, improved engagement and adherence, and reduced healthcare costs. Telehealth is being increasingly explored across community and home care to address a wide spectrum of health issues. Home health technologies play a crucial role in modern healthcare delivery, particularly for older adults. These technologies, used to monitor daily activities and manage health conditions, significantly enhance care and support daily tasks, thereby extending the ability of older adults to live independently at own home.^65,66^ Moreover, its application is being explored within the context of promoting equity, ensuring fair and equal access to services for all individuals, regardless of socioeconomic status, geographic location, race, ethnicity, gender, age, disparities in health, and digital literacy.^67,68^

Based on our review findings, telehealth enhanced specialized nurses’ involvement in home care yielded tangible outcomes for patient safety. Engagement is crucial in the work environment, as higher-level nurses’ involvement levels in care are linked to a lower incidence of patient safety issues.^69^ However, the complexity of care and healthcare organizational structures create challenging work situations and concerns emphasizing the need for guidelines and the standardization of professional practice.^70,71^ Also, nurses should acquire knowledge about the application of digital solutions in their practice field and actively participate in the development, use, and evaluation of digital health solutions to better address related safety concerns.^72–75^

Telehealth use by specialized nurses in our review addressed patient care concerns leading to heightened patient satisfaction with care, and consequently their feeling of safety with home care and confidence in the healthcare system. Generally, digital solutions emphasize prevention, placing patients at the center of care through patient empowerment, care personalization, precision, and interaction.^76^ Appropriate control and care optimization play pivotal roles in developing digital health initiatives through support for independent living by self-management, and timely interventions by remote monitoring and risk assessment.^77^

According to our review findings, telehealth improved specialized nurse-patient communication, collaboration, and empowered patients to adhere to their therapeutic regimens. However, one study highlighted the challenge of building patient relationships and maintaining privacy when using telehealth. The successful adoption of digital solutions into healthcare requires the consideration of individual patient's characteristics as users to prevent depersonalization and inequalities.^78^ Equity concerns, especially for at-risk populations’ access to digital technology and for digital literacy, should be addressed to ensure equal and inclusive access to essential healthcare services.^79^ Patients’ experiences encompass not just the technical aspects of a digital solution but also how it practically influences their daily life routines. Therefore, patient participation is required in the development and planned use of digital healthcare digital solutions as an integral component of maintaining and improving healthcare quality. It ensures that the design and implementation of healthcare technologies align with patients’ needs and preferences, fostering better user acceptance and usability.^80,81^

The challenges of integrating telehealth into specialized nursing practice in areas such as enhancing workloads and conducting thorough assessments were reported in our review. A common concern regarding the utilization of telehealth is the potential for an increased workload. Digitalization is an asset for public health, presenting potential benefits, which depend on factors like collegial and organizational support. There is a need for strategies to avoid undue stress, burnout, and potential compromises in the use of digital solutions.^82^ More research is needed to identify potential challenges that impact the integration of telehealth into the practice of specialized nurses in terms of technology limitations, privacy and security issues, communication challenges, and the need for training.

While our scoping review provides valuable insights, certain limitations inherent to this methodology should be acknowledged. Scoping reviews use a broad search strategy that can include studies of varying quality and methodologies, limiting the ability to draw specific conclusions or generalize findings across different contexts. This scoping review identified 23 studies published in the last decade examining specialized nurses’ roles in ensuring patient safety within the context of telehealth. The study only included original research articles that identified the roles of specialized nurses and does not reflect the broader use of telehealth by other health care workers, nor did it utilise material that pre-dates 2013. These restrictions may have hindered identification of potential concerns or pitfalls to be managed when integrating telehealth into the practice of specialized nurses. Language bias may have influenced this scoping review, which concentrated on studies published in English, possibly excluding pertinent research in other languages.^83^ Selection bias was avoided by encompassing studies from qualitative and quantitative research designs with a wide range of databases included in the search. By integrating qualitative findings, a more holistic view of the impact and effectiveness of telehealth interventions was provided. It allowed us to triangulate the data, ensuring that the findings were robust and reflective of both numerical trends and nuanced experiences.^84^ However, the variation of designs in the included set of articles might have impacted the data synthesis and integration. Further research, particularly using more rigorous systematic review methods, is needed to confirm and expand upon these findings.

## Implications of the research

Formulating evidence-based policies is essential to optimize the engagement of specialized nurses in utilizing telehealth for enhanced patient safety. It is imperative to develop tailored training programs that address their specific needs, focusing on proficiency in telehealth technologies and keeping them abreast of emerging advancements in this field. Ongoing education and collaboration among healthcare stakeholders are crucial for successfully integrating telehealth into nursing practice, thereby ensuring safer and more efficient patient care. These efforts will not only empower specialized nurses but also foster a healthcare environment that embraces innovative solutions for patient well-being. Conducting high-quality clinical trials with large sample sizes, along with robust modeling research, is crucial to bridging knowledge gaps and developing clinical guidelines. These efforts will support specialized nurses in effectively participating in safe care initiatives through digital solutions.

## Conclusions

According to our review findings, the integration of telehealth by specialized nurses led to multifaceted impacts on patient care in home care. It enhanced patients’ connectivity with healthcare providers, empowering them to actively manage their health through regular communication and adherence to follow-up plans. This proactive engagement improved overall satisfaction, feelings of safety, and confidence in the healthcare system. Furthermore, the use of telehealth facilitated early identification of potential health issues, enabling prompt interventions and preventing adverse events. Additionally, it effectively addressed concerns about healthcare costs leading to better adherence to therapeutic regimens.

Specialized nurses, through telehealth, played a crucial role in monitoring and enhancing medication management, significantly improving medication safety in home care. They achieved this by minimizing medication errors and addressing potential side effects and adverse drug reactions. The proficient use of digital solutions enabled specialized nurses to educate, engage, and empower patients, ultimately improving adherence to treatment plans and reducing the likelihood of adverse events.

## Supplemental Material

sj-doc-1-dhj-10.1177_20552076241287272 - Supplemental material for Specialized nurses’ role in ensuring patient safety within the context of telehealth in home care: A scoping review Supplemental material, sj-doc-1-dhj-10.1177_20552076241287272 for Specialized nurses’ role in ensuring patient safety within the context of telehealth in home care: A scoping review by Mojtaba Vaismoradi, John Rae, Hannele Turunen and Patricia A. Logan in DIGITAL HEALTH

sj-docx-2-dhj-10.1177_20552076241287272 - Supplemental material for Specialized nurses’ role in ensuring patient safety within the context of telehealth in home care: A scoping review Supplemental material, sj-docx-2-dhj-10.1177_20552076241287272 for Specialized nurses’ role in ensuring patient safety within the context of telehealth in home care: A scoping review by Mojtaba Vaismoradi, John Rae, Hannele Turunen and Patricia A. Logan in DIGITAL HEALTH

sj-docx-3-dhj-10.1177_20552076241287272 - Supplemental material for Specialized nurses’ role in ensuring patient safety within the context of telehealth in home care: A scoping review Supplemental material, sj-docx-3-dhj-10.1177_20552076241287272 for Specialized nurses’ role in ensuring patient safety within the context of telehealth in home care: A scoping review by Mojtaba Vaismoradi, John Rae, Hannele Turunen and Patricia A. Logan in DIGITAL HEALTH

sj-docx-4-dhj-10.1177_20552076241287272 - Supplemental material for Specialized nurses’ role in ensuring patient safety within the context of telehealth in home care: A scoping review Supplemental material, sj-docx-4-dhj-10.1177_20552076241287272 for Specialized nurses’ role in ensuring patient safety within the context of telehealth in home care: A scoping review by Mojtaba Vaismoradi, John Rae, Hannele Turunen and Patricia A. Logan in DIGITAL HEALTH
