# Supplementary material for: Specialized nurses’ role in ensuring patient safety within the context of telehealth in home care: A scoping review
Source: Digit Health. 2024 Oct 7;10:20552076241287272. doi: 10.1177/20552076241287272 (PMC11459674; doi:10.1177/20552076241287272)
Supplement: sj-doc-1-dhj-10.1177_20552076241287272 - Supplemental material for Specialized nurses’ role in ensuring patient safety within the context of telehealth in home care: A scoping review [file sj-doc-1-dhj-10.1177_20552076241287272.doc]

**Supplementary file 1.** Process of the scoping review: the specialized nurses’ roles in patient safety in the context of telehealth in home care

| **Aspect** | **Description** |
| --- | --- |
| **Reviewers** | MV, JR, HT, PAL |
| **Review aim/objectives/ questions** | Aim: To model the role of specialized nurses in ensuring patient safety within the context of telehealth in home care.  Explorative questions (corresponding to objectives) that in this review are sought to be responded are as follows:     - What knowledge gaps exist in the international literature concerning the role and responsibilities of specialized nurses in improving patient safety within the context of telehealth in home care? - What roles do specialized nurses have in the preservation of patient safety and prevention of errors within the context of telehealth in home care? - What are the indicators of preserving patient safety by specialized nurses within the telehealth context in home care? |
| **Review type** | A scoping review of the international literature, based on the Levac et al.'s (2010) framework, based on the Arksey and O'Malley methodology:  Levac D, Colquhoun H, O'Brien KK. Scoping studies: advancing the methodology. Implement Sci. 20 2010;5:69. doi:10.1186/1748-5908-5-69.  Arksey H, O'Malley L. Scoping studies: towards a methodological framework. *International Journal of Social Research Methodology*. 2005/02/01 2005;8(1):19-32. doi:10.1080/1364557032000119616. |
| **Language** | English |
| **Study designs** | All original research-based studies using qualitative, quantitative, and mixed-methods designs will be included.  Commentaries, letters, case reports, simulation studies that do not involve real patients in clinical practice, case studies and books lacking empirical data or are not aligned with the primary domains of this review will be excluded. |
| **PICO statement** | P (Population): Specialized nurses typically holding bachelor’s or master’s degrees in nursing and receiving additional training and education in a specific field of nursing to be equipped with specialized knowledge and skills to provide focused and expert care within a particular patient population or clinical setting. Examples are nurses formally recognized as advanced practice nurse (APN), clinical nurse specialist (CNS), nurse practitioner (NP), and clinical nurse consultant (CNC).  (Interest): Roles as practical considerations, interventions, and strategies to ensure patient safety in clinical practice.  Co (Context): Telehealth like video conferencing, mobile apps, and other digital platforms enabling virtual consultations, remote monitoring, and various healthcare activities, allowing the nurses to offer services, consultations, and support to distant patients living in their own home. |
| **Literature search** | Librarian assistance through consultation for the search process will be sought to ensure the accuracy of the process.    Electronic databases such as PubMed (covering MEDLINE), Scopus, CINAHL, Web of Science, ProQuest, and Embase will be searched for retrieving studies published from January 1, 2013, to August 29, 2024 using appropriate keywords.  Manual search and grey literature: Important journals publishing studies on digital health and telemedicine will be identified and searched.  Cross-references from the bibliographies of retrieved articles will be performed and current review papers, if any, for improving the search coverage will be considered. |
| **Literature selection** | Selecting all original studies based on titles, abstracts and full text of articles will be undertaken. The research team (MV, PL) will independently screen the retrieved studies and collaboratively will make decisions on their inclusion or exclusion based on the eligibility criteria.    The inclusion criteria for original and scientific content are as follows:  They should have focused on telehealth and navigated by specialized nurses in short-term, long-term, or community care settings for adults living in their own home receiving physical and mental healthcare. The studies should have acknowledged the elevated responsibilities designated to nurses, attributing to their classification as specialized nurses.  Studies centered on pediatrics, child, and neonatal care will be excluded due to the profound differences in clinical considerations that distinguish specialized nursing practices for them from adult care.  The search results will be shared with the review team members (MV, PL) via [rayyan.ai](https://www.rayyan.ai/). The team members will have access to retrieved studies for screening them for eligibility and making decisions for inclusion and exclusion. |
| **Research synthesis** | An extraction table will be drawn to chart data and import data from the selected studies and categorize their details.  An analytic framework will be developed by drawing tables to collate, summarize, and compare the studies' findings in relation to the review phenomenon. |
| **Equator guideline** | Customized JBI Critical Appraisal Tools will be used based on each article’s research design to evaluate studies:  JBI. JBI Critical Appraisal Tools 2020. Accessed August 2023. Http:/ <https://jbi.global/critical-appraisal-tools>.  The scoping review will adhere to the guidelines outlined in the Preferred Reporting Items for Systematic reviews and Meta-Analyses extension for Scoping Reviews (PRISMA-ScR):  Tricco AC, Lillie E, Zarin W, et al. PRISMA Extension for Scoping Reviews (PRISMA-ScR): Checklist and Explanation. Ann Intern Med. 2018;169(7):467-473. doi:10.7326/m18-0850. |
| **Funding sources/sponsors** | This research has received no funding from for conducting the review. |
| **Conflicts of interest** | The authors declare that the research is conducted in the absence of any commercial or financial relationships that could be construed as a potential conflict of interest. The authors preserve the copy-right to this article and keep the intellectual properties with regard to this study and all other ideas coming out of this study. |
