# Supplementary material for: Specialized nurses’ role in ensuring patient safety within the context of telehealth in home care: A scoping review
Source: Digit Health. 2024 Oct 7;10:20552076241287272. doi: 10.1177/20552076241287272 (PMC11459674; doi:10.1177/20552076241287272)
Supplement: sj-docx-2-dhj-10.1177_20552076241287272 - Supplemental material for Specialized nurses’ role in ensuring patient safety within the context of telehealth in home care: A scoping review [file sj-docx-2-dhj-10.1177_20552076241287272.docx]

**Supplementary file 2**. The list of keywords used for the search.

(“mobile health” OR “mHealth” OR “telehealth” OR “eHealth” OR “virtual medicine” OR “telehealth” OR “telemedicine” OR “digital health” OR “remote healthcare” OR “virtual care” OR “teleconsultation” OR “telemonitoring” OR “teletriage” OR “telehealth infrastructure” OR “telehealth technologies” OR “telehealth applications” OR “remote patient monitoring” OR “digital therapeutics” OR “wearable devices” OR “health monitoring devices” OR “Internet of Things (IoT) in healthcare” OR “artificial intelligence in telehealth» OR “machine learning in telemedicine” OR “virtual reality in healthcare” OR “augmented reality in healthcare” OR “telehealth policy” OR “telehealth regulations” OR “telehealth reimbursement” OR “telehealth adoption” OR “telehealth implementation” OR “telehealth effectiveness” OR “telehealth outcomes” OR “telehealth challenges” OR “telehealth privacy” OR “telehealth security” OR “telehealth ethics” OR “telehealth disparities” OR “telehealth in rural areas” OR “telehealth services” **AND** “patient safety” OR “safe care” OR “safe practice” OR “safe patient care” OR “mistake” OR “error” OR “medical error” OR “patient harm” OR “adverse event” OR “negligence” OR “violation” OR “malpractice” OR “failure” OR “mistreatment” OR “incident” OR “hazard” OR “near miss” OR “adverse health care event” OR “health care error” OR “harm” OR “danger” OR “healthcare quality” OR “medical error prevention” OR “patient harm reduction” OR “risk management in healthcare” OR “clinical safety” OR “patient protection” OR “adverse event prevention” OR “patient care safety” OR “preventable patient harm” **AND** Nurs* OR “nurse assistant” OR “nursing assistant” OR “nurse technician” OR “nursing technician” OR “registered nurse” OR “specialized nurse” OR “advanced practice nurse”, “emergency nurse practitioner” OR “nurse specialist” OR “clinical nurse specialist” OR “RN” OR “nurse practitioner” OR “licensed practical nurse” OR “LPN” OR “vocational nurse” OR “nurse clinician” OR “nurse manager” OR “nurse administrator” OR “nurse consultant” OR “nurse supervisor” OR “nurse informaticist” OR “public health nurse” OR “critical care nurse” OR “geriatric nurse” OR “home health nurse” OR “hospice nurse” OR “community health nurse” OR “palliative care nurse” OR “primary care nurse” OR “rehabilitation nurse” OR “gerontological nurse”)
