# Supplementary material for: Specialized nurses’ role in ensuring patient safety within the context of telehealth in home care: A scoping review
Source: Digit Health. 2024 Oct 7;10:20552076241287272. doi: 10.1177/20552076241287272 (PMC11459674; doi:10.1177/20552076241287272)
Supplement: sj-docx-4-dhj-10.1177_20552076241287272 - Supplemental material for Specialized nurses’ role in ensuring patient safety within the context of telehealth in home care: A scoping review [file sj-docx-4-dhj-10.1177_20552076241287272.docx]

**Supplementary file 4**. Summary of characteristics considered for the quality appraisal of the included studies in this scoping review

| Research type | Authors | Title | Role and level of education of the nurse | Method details: if QUAL –participant voice adequately represented; authors influence addressed; | Control Y/N | Philosophical approach congruent with methodology; question ; appropriate statistics | Identified confounding factors/Limitations/divergence between data in mixed-methods | Samples/Ethical approval indicated Yes/No | Outcome | Decision on inclusion |
| --- | --- | --- | --- | --- | --- | --- | --- | --- | --- | --- |
| Randomized controlled trial | Goldman et al., 2014 | Support from Hospital to Home for elders. A randomised Trial | NP undertook the intervention | Not disease related; looking at impact of intervention on re-admittance;  Participants not blinded to intervention,  Allocation to groups blinded to researchers – based on prev. 24hours admission list to ED | Y | Y | Y | Usual Care n = 352  *Vs.* Intervention (1on1 education + telephone follow-up) n = 347;  Randomised enrolment;  Ethics – grant supported but ethics approval not stated | No significant difference between groups | Yes |
| Randomized controlled trial | Vuorinen et al., 2014 | Use of Home telemonitoring to support multidisciplinary care of heart failure patients in Finland: Randomised Control Trial | Specialized HF nurses monitored patients weekly or more often f data was outside prescribed values and contacted when necessary | Telemetric data entered on mobile phone app weekly.  CHF patients | Y; Matched pairs | Y | Measured readmission and hospital days | Control group n = 47;  Telemonitoring group n = 47; Ethics - | No improvement in HF related hospital days; or patient clinical condition | Yes |
| Evidence-based practice project | Brainerd and Hawkins, 2016 | Enrollment of veterans into a heart failure home telehealth program | HT nurses - case managers;  HT nurses reviewed telemetric data daily and did patient contacts; referred on the APN on occasion | Protocol to improve enrolment in telehealth program with the intention to reduce re-admissions | N | Y | Small study | n=24 at study end;  n=99 referred with 32 enrolled in HT program – 8 were withdrawn over time;  Ethics – Y | Positive impact on readmissions and telehealth enrollments | Yes |
| Randomized controlled trial | Ishani et al., 2016 | Telehealth by interprofessional team in patients with CKD. An RCT | APN - telemonitoring & Medical management in consult with clinical pharmacist & virtual visits | Intervention: in home telemonitoring and telephone contacts for discharged CKD patients;  Computer generated randomisation to groups | Y | Y | Did not track procedure types for usual care group – only the intervention group; short study | Intervention (telemonitoring) n = 450  *Vs.*Usual Care n = 150  Randomised enrolment;  Ethics – funded but not stated | No significant difference between groups (secondary endpoints or hospitalisations | Yes |
| Pilot intervention | Moore, 2016 | Evaluation of the efficacy of a Nurse Practitioner-led home-based congestive heart failure clinical pathway | NPs role in HF | Telemonitoring intervention Admitting RN trained patients in equipment use, telemonitoring occurred daily -alerts provided to NP for triage; they virtual visit with patients each week  Convenience sample.  Impact on readmission rates.  Pre-post admission rates | N | Y  Descriptive statistics | Limitation of access to qualified NPs particularly across borders in US requiring multiple licenses. | n=22 with 27 visits and 26 NP interventions | Positive impact on readmission rates | Yes |
| Pilot study | De Heide et al 2017 | A Pilot feasibility study of telemedical wound assessment using mobile phone in cardiology patients. | NP x 2 evaluated all wound pictures | Inter-operator assessment - agreement of postoperative evaluation of wounds using images supplied by patients remotely  Images de-identified | N | Y | Evaluation – subjective;  Image quality  Assessment: not completed in real-time | n= 40    Ethics - Y | Successful pilot | Yes |
| Prospective pilot cohort | Fisher et al., 2019 | Development of an entirely remote, non-physician led hypertension management program | NPs and Pharmacists informed the algorithm devel. And trained the patient navigators | This is a description of the implementation of a pilot remote monitoring program (algorithm) and the lessons learned in developing it. Pharmacist adjusted Medications. | N | Data analysed was the monitoring data with patient demographics and medical notes | No long term clinical outcome data.  Pilot data | n=130; Ethics – N statement | Positive outcomes for blood pressure control | Yes |
| Cross sectional study | McNeal et al., 2019 | Applying Person-centred principles to the design and implementation of a virtual nurse managed clinic | APC are primary care givers in the clinic. | Looks at cost implications, loss to follow-up, and demographic data to determine if clinic reaching those it was intended to reach, patient insurance cover | N | Y |  | n=477 treated by APNs;  Ethics - Y | Successful project | Yes |
| An observational, pre-post, multimethod, and triangulation | McGloin et al., 2020 | Patient empowerment using electronic telemonitoring and telephone support in the transition to insulin therapy in Adults with type II diabetes | Clinical Nurse Specialist monitored the telemonitoring data; contacted patients when needed and triaged;  CNS conducted the focus groups with patents and follow-up at 3 months | 12 week telemonitoring and telephone support program;  Convenience sampling | N | Y | Confounders: was positive outcome due to monitors *or* telephone support *or* both | n = 39;  Ethics – Y | Positive outcome | Yes |
| Intervention | Ruf et al., 2020 | Videoconference clinics improve efficiency of inflammatory bowel disease care in a remote and rural setting. | inflammatory bowel disease (IBD) specialist nurses operating a virtual IBD clinic | Looked at consistency of use of the VC appointments and savings made based on distance travelled (Patients were from remote Scottish areas) and patient outcomes;  Note VC clinics were in medical facilities that did not have IBD services not patient homes | N | Y | Outcomes resulted in recommended move to a full trial that includes a control group | n= 88 patients with n=229 appointments assessed | Good outcome - 92% did not participation in VC clinic appts;  Patient Travel costs saved $US36.61 per appt. based on NHS reimbursement rates | Yes |
| Qualitative | Seuren et al., 2020 | Physical examinations via a video for patients with heart failure qualitative study using conversation analysis | Specialist Nurse-led video examination of patient in presence of carer to assist. | Video recordings of with community specialist nurses – micro conversation analysis | N | - | - | n=7; Ethics: Consent for recording provided |  | Yes |
| Intervention | de Peralta et al., 2021 | Leveraging telemedicine for management of veterans with heart failure during COVID-19 | NP led program HF virtual care and triage for ED when required (phone or video) | COVID resulted in conversion of F2F visits to virtual visits -either by phone or video. Study period was 8 weeks – respiration, oedema, pallor and JVP and medications were all able to be assessed via video with carer assistance | N | Y   Descriptive stats | Y  Using appointment data and reflective practice – essentially a description of what they did | NPs conducted 132 visits to 132 patients with 3 patients referred to ED;  Ethics – not stated | Positive outcome; increased use of video by end of study period | Yes |
| Randomized controlled trial | Ho et al., 2021 | Testing the feasibility of Sensor-based home health monitoring (TEC4Home) to support the convalescence of patients with heart failure: Pre -Post study | Monitoring was completed by a single cardiac specialty nurse with 20 years cardiac experience. – triaged with HF NPs and other specialists | Testing the TEC4Home  - pre and post patient use surveys plus a number of health survey scales eg QoL, EHFScBs-9 selfcare behavior scale etc.  Adherence to protocol, outcomes, nurse interview | N | Y | n= 70, Ethics - Y |  | Positive outcome led to RCT | Yes |
| Mixed-methods process evaluation of a pilot | Indraratna et al 2021 | Process Evaluation of a Randomised Control Trial for TeleClinical Care, a Smartphone-app based model of care. | HF specialist nurses were involved in HF management protocol; HFOS (HF Outreach Service) is managed by a NP and CNS | Data: Interviews with NP, CNS, GPs and Cardiologists + cardiac rehab completion rates + outreach service referrals + specialist follow-up | N | Y | Smartphone ownership or incompatible smartphone limited enrolment in RCT | Ethics – Y | Positive outcome | Yes |
| Feasibility pilot intervention | McLachlan et al., 2021 | An NP-led pilot telehealth programme to facilitate guideline-directed medical therapy for heart failure with reduced ejection fraction during the COVID-19 pandemic | NP or CNS supported patients to monitor vital signs, and identify fluid congestion. Monitor medication titration. | Convenience sample Descriptive stats | N | Y | Non English speaking patients excluded; group of patients recruited may not reflect entire HF population; small feasibility study; mobile phone access inconsistent. | n=50 | Positive outcome – proceed to full study | Yes |
| Pilot intervention | Maher et al., 2022 | Virtual clinic for positive fecal occult blood tests FOBT enhances early access to bowel cancer screening and is well received by patients | Clinical nurse consultant completed pre screening and post colonoscopy virtual visits and undertook DAC bookings to colonoscopy | Measuring impact on wait times using a nurse led protocol for pre colonoscopy assessment following positive FOBT, colonoscopy booking and post surgery virtual visit;  Not random – based on a single practice and referral pathway;  VC model survey completed by patients – simple descriptive stats | Y – control group was those referred through usual pathway retrospective data | Y |  | n=167 patients of which 130 went to DAC and 5 triaged to out-patient clinic by CNC; Normal referral pathway n = 49;  Ethics – Y | Positive impact on wait times ie wait times to colonoscopy decreased;  majority indicated virtual clinics were a positive experience | Yes |
| Prospective study | Nehme et al., 2023 | Study of prehospital video telehealth for callers with mental health-related complaints | MHNs – located in EMS call centre undertook triage for those with mental health issues | Outcomes, cost analysis, and MHN interviews and patient follow-ups and surveys | N | Y  MHN voice not in data; who did the interviews not identified; Impact of interviewer not outlined |  | n=738 MHN consultations via video | Video telehealth required less EMS dispatches than voice only by non-MHN triage. | Yes |
| Randomized trial | Prescher et al., 2023 | Patient reported experience and adherence to remote patient management in chronic heart failure patients: a posthoc analysis of the TIM-HF2 trial. | HF specialist nurses completed device installation, Patient training with devices, and chronic disease self-management and any additional training by phone and contacted patients once per month | Post trial survey to RPM group; Post hoc Protocol adherence | N | Y | 26 HF nurses managed the patients | n=796 randomly assigned to remote monitoring RPM group in original trial - of whom 564 did the survey - Ethics- Y | Protocol considered acceptable. | Yes |
| Pilot randomized trial | Schmaderer et al., 2023 | Feasibility, acceptability, and intervention description of a mobile health intervention in patients with heart failure | MHealthPlus  Group were provided with virtual visits by a Cardiac NP/community health worker team | Pilot study: randomised 3 group repeated measure design. App evaluation; App was designed to facilitate self-care; | Y | Y | Not clear if virtual visits added any significant difference to outcomes of using the app;  statistics descriptive. | Usual care  n=27  mHealth app  n=26  MHealthPlus  n=2 – Ethics- Y | Positive outcome for both intervention groups | Yes |
| Intervention | Zhang and Guo, 2023 | The effect of internet-based telehealth nursing on the quality of life in patients with atrial fibrillation and stroke | 3 nurse stroke specialists in cardiovascular medicine and 3 stroke nurse specialists in neurology | Telehealth nursing vs. standardized early-stage rehabilitation included health education and caregiver training before discharge, post-discharge by community and follow-up | Y | Y | Y | 168 patients with atrial fibrillation and stroke: 84 in the internet-based telehealth nursing group (audio and video calls, while patients used ECG and blood pressure monitors) and 84 in traditional at-home self-help nursing group- Ethics- Y | Survival rate, readmission rate, daily activities, limb motor ability, psychological state, cardiac function, rhythm control, adverse events, and nursing satisfaction | Y |
| Qualitative | Anderson et al., 2024 | Remote and technology-mediated working during the COVID-19 pandemic: A qualitative exploration of the experiences of nurses working in general practice (the GenCo Study) | 8 ANPs performed video consultations and telephone follow up | Remote, technology-based nursing care was provided during the COVID-19 waves | N | Y | - | 8 ANPs – Ethics- Y | Issues of technology access, workload, hybrid work, disrupted relationships, safety risks, decision-making exclusion | Yes |
| Prospective, international, multicentre, single-arm interventional study | Leenen et al., 2024 | Usability of a digital health platform to support home hospitalization in heart failure patients: a multicentre feasibility study among healthcare professionals | Specialist HF nurses (18 cardiac care nurse, 1 NP) conducted daily home visits to monitor patients, take blood samples, and adjust IV drug therapy as needed, staying in close contact with cardiologists. Patients also received the Digital Health platform. | The digital platform features a blood pressure device, weight scale, pulse oximeter, wearable chest patch, and an eCoach app with educational videos and daily symptom checks for HF | N | Y | Y | 63 Patients with acute decompensation of pre-existing HF, well-assessed chronic HF – Ethics- Y | Perceived acceptability, appropriateness, feasibility, and satisfaction | Yes |
| Single-site, pilot randomized controlled trial | Mirshahi et al., 2024 | The impact of an integrated early palliative care telehealth intervention on the quality of life of heart failure patients: a randomized controlled feasibility study | 1 nurse interventionist Led a six-week virtual program on palliative care for heart failure patients, featuring weekly webinars and follow ups | The feasibility and acceptability of a telehealth palliative care intervention aimed at improving quality of life in HF patients | Y; control group patients via WhatsApp® (n=25) | Y | Y | 50 patients: 25 in each group- Ethics- Y | Feasibility, acceptability, quality of life, satisfaction, attrition, anxiety, depression, visit to the ED | Yes |

ED: emergency department; HT: home telehealth; HF: heart failure; CHF: chronic heart failure; RCT: randomized controlled trial; CKD: chronic kidney disease; NP: nurse practitioner; RN: registered nurse; APC: advanced practice nurse; ANP: advanced nurse practitioner; CNS: clinical nurse specialist; IBD: inflammatory bowel disease; VC: virtual clinic; NHS: national health service COVID: coronavirus disease 2019; JVP: jugular venous pressure; GP: general practitioner; QoL: quality of life; HFOS: HF outreach service; DAC: direct access; EMS: emergency medical services; MHN: mental health nurse; RPM: remote program management; ECG: electrocardiogram; IV: intravenous
